# Online Health Information Seeking and Preventative Health Actions: Cross-Generational Online Survey Study

**DOI:** 10.2196/48977

**Published:** 2024-03-11

**Authors:** Jayati Sinha, Nuket Serin

**Affiliations:** 1 Department of Marketing & Logistics College of Business Florida International University Miami, FL United States; 2 W. Fielding Rubel School of Business Bellarmine University Louisville, KY United States

**Keywords:** digital natives, digital immigrants, online health information seeking, preventative health actions, mobile phone

## Abstract

**Background:**

The popularity of online health information seeking (OHIS) has increased significantly owing to its accessibility and affordability. To facilitate better health management, it is essential to comprehend the generational differences in OHIS behavior and preventative health actions after seeking online health information (OHI).

**Objective:**

This study investigates the variations in OHIS and engagement in preventative health actions between 2 generations based on their technology use (digital natives [aged 18-42 years] and digital immigrants [aged ≥43 years]). Additionally, this research explores the mediating role of OHIS types on the generational effect on preventative health actions and the moderating role of OHI search frequency, gender, and the presence of chronic diseases on the generational effect on OHIS types and preventative health actions.

**Methods:**

A preregistered online survey was conducted on the Prolific online data collection platform using stratified sampling of 2 generations (digital natives and digital immigrants) from the United States in November 2023. Overall, 3 types of OHIS were collected: health wellness information search, health guidance information search, and health management information search. A 1-way analysis of covariance tested the generational differences in types of OHIS and preventative health actions, and a 2-way analysis of covariance tested the moderating role of OHIS search frequency, gender, and the presence of chronic diseases using 7 control variables. The PROCESS Macro Model 4 was used to conduct mediation analyses, testing OHI search types as mediators. Linear regression analyses tested age as a predictor of OHIS and preventative health actions.

**Results:**

The analysis of 1137 responses revealed generational differences in OHIS. Digital natives searched for health wellness information more frequently (*P*<.001), whereas digital immigrants searched for health guidance (*P*<.001) and health management information (*P*=.001) more frequently. There were no significant differences between the 2 generations regarding preventative health actions (*P*=.85). Moreover, all 3 types of OHIS mediated the relationship between generational differences and preventative health actions. Furthermore, as people aged, they searched for significantly less health wellness information (*P*<.001) and more health guidance (*P*<.001), and health management information (*P*=.003). Age was not a significant predictor of preventative health actions (*P*=.48). The frequency of OHI searches did not moderate the effect of generations on OHIS types and preventative health actions. Gender only moderated the relationship between generation and health guidance information search (*P*=.02), and chronic diseases only moderated the relationship between generation and health wellness information search (*P*=.03).

**Conclusions:**

To the best of our knowledge, this study is the first to explore how 2 digital generations vary in terms of searching for OHI and preventative health behaviors. As the older adult population grows, it is crucial to understand their OHIS behavior and how they engage in preventative health actions to enhance their quality of life.

## Introduction

### Background

Online health information seeking (OHIS) has become popular owing to its convenience and low-cost access [1]. Although the internet remains a popular source of health information [1], an age-based digital divide exists in OHIS behavior—young generations (such as Millennials) are more likely to seek online health information (OHI) than older generations (such as Baby Boomers) [2-7]. The gap between younger and older Americans has widened in recent years—the number of Americans aged ≥55 years grew by 27% between 2010 and 2020, whereas the number of Americans aged <55 years increased by only 1.3% during that time [8]. As the US population ages, the demand for health care and medical information increases for older generations [1,7]. However, older generations’ OHIS behavior, specifically types of health-related information searches, is not well understood [9], and it is unclear to what extent older and younger Americans engage in preventative health behaviors after seeking OHI [9-11]. Moreover, the increased availability of online medical information has made patient-centered medical care more common, allowing health consumers to make informed decisions about their health. Thus, understanding the generational differences in OHI search behavior and subsequent health-related actions is crucial for designing more effective communication methods to improve and maintain health. This study explored how 2 generations with different experiences with technology (digital natives and digital immigrants) differ in their OHI search activities and preventative health actions.

### Digital Natives and Digital Immigrants

Generations are defined by the years of birth. Generational cohorts are groups of people who share similar cultural, historical, and technological experiences growing up and tend to develop similar beliefs, values, and behaviors as they age [12,13]. Generations are often defined by how they use and grow up with technology [14]. Indeed, the Technology Model of Generations [15] suggests that technology is the root cause of most generational differences and indirectly affects other sociocultural forces that shape each generation (such as a shift from collectivism to individualism and the introduction of a slow life strategy). Prensky [16] defined 2 generational groups based on their preferences for technology or digital devices: digital natives (born from 1980 onward) and digital immigrants (born before 1980). Digital natives were raised in a technology-driven world (such as smartphones, tablets, and laptops as commonplace items in daily life), whereas digital immigrants did not have the same experiences with technologies [15-20].

Exposure to digital environments (or lack thereof) affects individuals’ use, fluency, comfort level, literacy, and engagement with technology [21-25] as well as how they think and behave [26,27]. Digital natives tend to process digital information quickly, multitask more, and depend on technology to communicate and access information and are usually more comfortable with technology than their digital immigrant counterparts [28,29]. As digital technologies have been integrated into the lives of digital natives since the early days, they feel more at ease with OHI searches than digital immigrants [30,31]. Indeed, nearly 48% of Americans aged <30 years use the internet constantly compared with 22% of Americans aged 50 to 64 years and 8% of Americans aged >65 years [32].

Researchers have suggested studying 2 generations along a continuum based on user technology proficiency, digital literacy, or engagement with technology rather than the age-based classification of digital natives and digital immigrants [22,25]. Although differences in computer efficacy, socioeconomic status, health literacy, and technology accessibility might help explain some differences between digital natives and digital immigrants [33-35], a generation gap exists in attitude and behavior toward technology use [16,17]. Research shows that younger people prefer the internet as a source of health information [3,7,36], and with increased age, the information processing capacity for technology-enabled tasks diminishes [29]. Previous research has shown that negative attitudes toward internet use as well as privacy concerns about using technology decrease older adults’ OHIS behavior [9,35]. Given that digital natives grew up with digital technology and are familiar with it from a young age, their OHIS behavior is likely to differ from that of digital immigrants as each generation uses technology differently [32]. Accordingly, digital natives and immigrants may differ in their search for various types of OHI and engagement in preventive efforts after retrieving such information. Thus, this study focused on 2 generations that differ in their level of technology immersion: digital natives and digital immigrants [16].

### Seeking OHI and Preventative Behaviors

Health consumers seek OHI for various reasons, including gaining knowledge about health issues, managing health problems, and making health decisions [37-39]. Research has shown that OHI seekers frequently search for disease-related information [40]. In addition, people seek information about symptoms, medication, treatment, exercise and fitness, and nutrition or diet [41-43]. However, only a few studies have examined what types of OHI older and younger generations seek [9,43,44]. For example, Chinese OHI seekers most often search for information about health science popularization to improve their health literacy, whereas medical concerns are the least explored type of health information [43]. A recent review of the literature on health information seeking indicates that older adults seek various types of OHI, including information about specific diseases, medications and treatments, disease symptoms, nutrition and exercise, medical resources, and interpersonal advice [9]. Compared with older adults, adolescents and young adults seek online information on physical and psychological well-being, sexual health, and culturally sensitive topics [9]. In addition, OHI searches are lower for German and Australian older adults aged >65 years [7,36]. Moreover, young adults seek specific health information online by joining social media groups or following relevant pages [44]. It has also been found that the frequency of OHIS is higher among female individuals [43,45,46] and younger people [3,7,36], whereas people with chronic diseases are more likely than healthy people to perform OHI searches [47,48]. Although racial disparities exist in OHIS behaviors [49-51], not all racial groups seek out information equally, and chronic diseases have been found to be more important than race or ethnicity in OHI search and use [52]. Moreover, political affiliation is associated with various health outcomes, including health information searches, vaccine adoption, and preventative health behaviors [53-56].

Despite the availability of a large amount of health information online [57], some OHI can be inaccurate or misleading, causing unnecessary distress and anxiety among health consumers [58,59]. The inaccuracy of OHI raises concerns about its quality [60]. For example, health consumers, especially young people, increasingly rely on social media as their first resource for health information. However, online content is often unverified and can be misleading [61,62]. Specifically, research has shown that older adults tend to be concerned about the credibility of OHI sources [63]. Moreover, low health literacy prevents people from accurately determining the credibility of OHI on social media [34,35,64,65]. In addition, health consumers’ trust in OHI is associated with their self-efficacy beliefs and behavioral outcomes [66], and their confidence in OHIS skills is positively related to the use of the retrieved information [67]. Previous studies have identified that credibility, usefulness, and trust most frequently affect older adults’ OHIS and health behaviors [9,39].

Access to OHI has become critical for managing health and disease, and it generally leads to positive outcomes such as improved health outcomes and empowered patients [68,69]. Research shows that health information seeking improves patient involvement and satisfaction with medical decision-making and communication between patients and informal care providers [70,71]. Despite the potential of OHIS to affect health behaviors across different age groups [47,72], little research has examined whether different generations based on their technology adoption engage in preventative behaviors after seeking OHI in the same way. As a result, there is a clear need to understand what types of health information different digital generations seek online [9,11]. Thus, this study focused on generational differences based on the use of technology or digital devices in seeking out types of health information online and engaging in preventative health actions afterward.

### Objectives

This study addressed the limitations of previous research on information seeking from a generational perspective. It focused on 2 generations that differ in technology proficiency (digital natives and digital immigrants) to examine how they differ in their OHI search behavior. This study aimed to determine the types of OHI that digital natives and digital immigrants seek as well as whether each generation varies in their engagement in preventative health actions after seeking different types of OHI. The following three research questions guided this study:

1. How do generational differences influence different types of OHIS and preventative health actions?
2. Do types of OHIS behaviors mediate the generational effect on preventative health actions?
3. Do the frequency of OHI searches, gender, or the presence of a chronic disease moderate the generational effect on OHIS types and preventative health actions?

## Methods

### Study Design

We conducted an online survey to understand how OHIS behavior influences preventative health behaviors across 2 generations (digital natives and digital immigrants) and whether the frequency of OHI searches, gender, and the presence of a chronic disease moderate the effect. We used a 2-factor mixed factorial design where the first factor was a between-subject moderator (2 digital generations: natives vs immigrants) and the second factor was a within-subject moderator (frequency of OHI searches [frequent vs infrequent], gender [male vs female], or presence of a chronic disease [yes vs no]).

### Ethical Considerations

The Bellarmine University Institutional Review Board (IRB) approved this study under the *exempted research* category (IRB 1092) before data collection, and general information about the nature of this study was included at the beginning of the survey as a means of informed consent. No formal informed consent was obtained. This study was preregistered on the AsPredicted platform (Wharton Credibility Lab) [73]. The online platform Prolific was used for data collection. Researchers using Prolific only had access to a user ID, and the respondents’ identities were not revealed. An online survey was designed using the Qualtrics survey design software (Qualtrics International Inc), which did not capture any identifying information. We provided respondents with a monetary reward (US $0.40) as an incentive for participation.

### Data Collection

Study data were collected in November 2023 from Prolific, an online data collection platform, using samples of adults from the United States. We selected Prolific as an online data collection platform because of its high data quality compared with Amazon Mechanical Turk and CloudResearch [74]. We used a power analysis using the G*Power software (version 3.1; Heinrich-Heine-Universität Düsseldorf) to identify the appropriate sample size [75]. The power analysis results revealed that, for a small (0.15) effect size with α=.05 and a statistical power of 0.95, the total minimum sample size was approximately 768 respondents (4 groups and 7 covariates). We used a similar stratified sampling method [76] based on age, sex, and political affiliation to understand the OHIS behavior of adults in the United States. Specifically, we aimed to collect an age-, sex-, and political affiliation–stratified sample of 1200 respondents to ensure an adequate sample size after eliminating respondents based on the data exclusion criteria outlined in the preregistration. On the basis of the age and US political affiliation criteria, we aimed to recruit 300 digital natives (aged between 18 and 42 years) of Democratic affiliation, 300 digital natives of Republican affiliation, 300 digital immigrants (aged between 43 and 99 years) of Democratic affiliation, and 300 digital immigrants of Republican affiliation from Prolific for this study (a total of 1200). To ensure a sample representative of both sexes, we used *balanced sample* criteria for an even distribution of male and female respondents. Thus, an age-, sex-, and political affiliation–stratified sample was used to ensure an equal number of respondents in the 2 digital generation groups (digital natives vs digital immigrants), with an even distribution of gender (male and female) and political affiliation (Republican and Democratic). In addition, we recruited respondents from the United States using *location* criteria as well as those with a 100% approval rate from Prolific to ensure data quality. The final recorded responses were from 299 digital immigrants who were Democrats, 311 digital immigrants who were Republicans, 310 digital natives who were Democrats, and 309 digital natives who were Republicans (a total of 1229 responses were used for the analysis after merging 4 surveys). This deviation was due to the Prolific software and was outside the researchers’ control.

### Data Exclusion

On the basis of the exclusion criteria outlined in the preregistration, we excluded 92 respondents in the following order—17 (18%) failed the attention check question, 8 (9%) did not complete the survey, 26 (28%) responded “never” for the frequency of OHIS, and 7 (8%) participants’ age and 34 (37%) participants’ political affiliation did not match for the respective surveys—from the sample, leaving 1137 (n=571, 50.22% digital natives and n=566, 49.78% digital immigrants) responses for analysis. The operationalization and coding of the focal study variables are shown in Table 1.

**Table 1:** The operationalization and coding of study variables.

Variables	Operationalization and coding	References
Generation	Digital natives=aged between 18 and 42 years; digital immigrants=aged ≥43 years	Ransdell et al [18], 2011; Roth-Cohen et al [77], 2021
Frequency of OHIS^a^	“How frequently did you search for online health information in the past 12 months?” (“frequent,” “infrequent,” and “never”; 1=frequent; 2=infrequent; respondents who selected “never” were excluded)	Xiong et al [43], 2021
HWIS^b^	The sum of 3 types (sports and fitness, nutrition and diet, and general health knowledge; 1=yes; 0=no)	Neter and Brainin [78], 2012; Xiong et al [43], 2021
HGIS^c^	The sum of 2 types (medication guidance and disease consulting; 1=yes; 0=no)	Neter and Brainin [78], 2012; Xiong et al [43], 2021
HMIS^d^	The sum of 2 types (managing health conditions and participating in an online support group; 1=yes; 0=no)	Neter and Brainin [78], 2012; Xiong et al [43], 2021
PHA^e^	The sum of 3 items (“After seeking health information and finding the information, did your health behavior change for the better? After seeking health information and finding the information, did you see a doctor? After seeking health information and finding the information, did you monitor your health yourself for any changes?”; 1=yes; 0=no)	Taylor and Humphrey [79], 2022
Gender	1=male; 2=female	N/A^f^
Marital status^g^	1=married; 2=single	Xiong et al [43], 2021
Employment^h^	1=unemployed; 2=employed	Xiong et al [43], 2021
Education level	1=high school graduate or lower; 2=high school graduate; 2=some college or associate degree; 4=bachelor’s degree; 5=graduate degree or higher	Gordon and Crouch [80], 2019
Income	1=<US $25,000; 2=US $25,000-$34,999; 3=US $35,000-$49,999; 4=US $50,000-$64,999; 5=US $65,000-$79,999; 6=US $80,000-$99,999; 7=≥US $100,000	Gordon and Crouch [80], 2019
Race	1=White; 2=Black or African American; 3=American Indian or Alaska Native; 4=Asian; 5=Native Hawaiian or other Pacific Islander; 6=some other race	Jensen et al [81], 2021
Ethnicity	1=Hispanic or Latino; 2=not Hispanic or Latino	Jensen et al [81], 2021
Political affiliation	1=Democrat; 2=Republican; 3=independent; 4=other	Naeim et al [55], 2021

^a^OHIS: online health information seeking.

^b^HWIS: health wellness information search.

^c^HGIS: health guidance information search.

^d^HMIS: health management information search.

^e^PHA: preventative health actions.

^f^N/A: not applicable.

^g^Marital status was divided into 2 categories: single (unmarried, divorced, or widowed) and married.

^h^Employment was recorded as 2 groups: unemployed (unemployed, students, and retired) and employed.

### Study Measures

We adapted the online survey measures based on previous research. Multimedia Appendix 1 contains a list of all the measures in the order in which they appeared in the survey.

#### Digital Generations: Natives and Immigrants

On the basis of previous research [18,77], we operationalized 2 digital generations based on age: digital natives (Generation Z and Millennials; aged between 18 and 42 years) and digital immigrants (Generation X and Baby Boomers; aged ≥43 years).

#### Frequency of OHIS

The frequency of OHIS was examined using a subjective assessment of how frequently respondents engaged in OHIS in the previous 12 months on a 3-point scale (*frequent*, *infrequent*, and *never*; adapted from the study by Xiong et al [43]). We excluded respondents who selected *never*. Those who chose *frequent* were grouped as frequent OHI seekers, and those who indicated *infrequent* were grouped as infrequent OHI seekers.

#### Health-Related Information

For health-related information, we asked respondents the following: “Please indicate whether you have searched for the following health-related information during the past 12 months.” We presented 7 types of health-related information with a dichotomized (*yes* or *no*) response (adapted from the studies by Xiong et al [43] and Neter and Brainin [78]). We grouped them into 3 search categories: health wellness information search (HWIS; sports and fitness, nutrition and diet, and general health knowledge), health guidance information search (HGIS; medication guidance and disease consulting), and health management information search (HMIS; managing health conditions and participating in an online support group). We created 3 scores by adding the responses in the 3 search categories (HWIS, HGIS, and HMIS).

#### Preventative Health Actions

Preventative health actions were measured using 3 items with a dichotomized (*yes* or *no*) response (“After seeking health information and finding the information, did your health behavior change for the better? After seeking health information and finding the information, did you see a doctor? After seeking health information and finding the information, did you monitor your health yourself for any changes?”; adapted from the study by Taylor and Humphrey [79]). We computed a preventative health actions score by summing the responses to the 3 items.

#### Control Variables

We tested 7 control variables. We controlled for information quality, trust, and search skills (adapted from the studies by Miller and Bell [30], Diviani et al [60], and Xiao et al [82]): the *quality* of OHI (“How would you rate the quality of health-related information on the Internet?” [1=*very good quality*; 7=*very poor quality*]), *trust* in OHI (“How much would you trust health-related information on the Internet?” [1=*a lot*; 7=*not at all*]), *trust* in US health care institutions (“How much would you trust healthcare institutions in the US?” [1=*a lot*; 7=*not at all*]), and respondents’ online health-related information *search skills* (“Please rate your health-related information search skills on the Internet.” [1=*very good*; 7=*very poor*]). In addition, we used 3 other control variables: *race* (“What is your race?” [*White*, *Black or African American*, *American Indian or Alaska Native*, *Asian*, *Native Hawaiian* *or other Pacific Islander*, and *some other race*]; adapted from the study by Jensen et al [81]), *ethnicity* (“What is your ethnicity?” [*Hispanic or Latino* and *not Hispanic or Latino*]; adapted from the study by Jensen et al [81]), and *political affiliation* (“What is your political affiliation?” [*Democrat*, *Republican*, *independent*, and *other*]; adapted from the study by Naeim et al [55]). We used these 3 variables as covariates in all analyses.

#### Sociodemographic Information

Demographic information included age, gender, annual household income, education, marital status, and employment status. We also asked respondents whether they went for a regular annual checkup (“Do you go for annual checkup regularly?” [*yes* or *no*]), whether they had health insurance (“Do you have health insurance?” [*yes* or *no*]), and whether they had at least one chronic disease (“Do you have at least 1 chronic disease?” [*yes* or *no*]), and descriptive statistics were reported for these measures.

#### Attention Check

To test whether respondents paid attention while completing the online survey, we asked the following: “If you are reading this, please do not answer this question and leave it blank.” (1=*not at all true of me*, 2=*slightly true of me*, 3=*moderately true of me*, 4=*very true of me*, and 5=*extremely true of me*), adapted from the study by Chugani and Irwin [83]. We excluded respondents who failed the attention check.

### Statistical Analysis

We used SPSS (version 29; IBM Corp) to analyze the data. We conducted 5 sets of statistical analyses outlined in the preregistration while controlling for 7 factors (quality of OHI, trust in OHI, trust in health care institutions, OHI search skills, race, ethnicity, and political affiliation). Specifically, we conducted 4 one-way analyses of covariance (ANCOVAs) using generation as an independent variable, 3 types of health information searches (HWIS, HGIS, and HMIS) and preventative health behavior (preventative health actions) as dependent variables, and 7 covariates. In addition, to test the effect of age (a continuous measure instead of 2 generations) on OHIS types (HWIS, HGIS, and HMIS) and preventative health actions, we performed 4 linear regression analyses using age as an independent variable, OHIS types (HWIS, HGIS, and HMIS) and preventative health actions as dependent variables, and 7 covariates.

To test whether search frequency moderated the effect of generation, we conducted 4 two-way ANCOVAs using generation and search frequency as independent variables, the 4 focal variables (HWIS, HGIS, HMIS, and preventative health actions) as dependent variables, and 7 control variables. To test whether gender moderated the effect of generation on the same 4 focal variables, we conducted 4 two-way ANCOVAs using generation and gender as independent variables and 7 control variables. In addition, to explore whether the presence of chronic diseases moderated the effect of generation on the 4 focal variables, we conducted 4 two-way ANCOVAs using generation and the presence of chronic diseases as independent variables and 7 control variables.

To test the types of health information searches as mediators, we used the bootstrapping approach as applied in the SPSS PROCESS (version 3.4) macro [84]. We also performed a descriptive analysis of sociodemographic characteristics.

## Results

### Sociodemographic Characteristics of Respondents

Table 2 presents the detailed sociodemographic characteristics of the study sample by generation (digital natives and digital immigrants). The study sample comprised 49.52% (563/1137) female individuals and had an average age of 44.2 (SD 13.8) years. Most respondents were single (614/1137, 54%) and employed (838/1137, 73.7%). Approximately 31% (351/1137, 30.87%) of respondents had some college education or associate degrees, 13.1% (149/1137) were high school graduates, 40.37% (459/1137) had bachelor’s degrees, and 15.04% (171/1137) had graduate degrees or higher. Approximately 11.96% (136/1137) of respondents earned <US $25,000 a year, 9.32% (106/1137) earned between US $25,000 and US $34,999 a year, 15.04% (171/1137) earned between US $35,000 and US $49,999 a year, 12.31% (140/1137) earned between US $50,000 and US $64,999 a year, 11.79% (134/1137) earned between US $65,000 and US $79,999 a year, 10.91% (124/1137) earned between US $80,000 and US $99,999 a year, and 28.67% (326/1137) earned ≥US $100,000 a year. Most respondents reported being White (931/1137, 81.88%) and of non-Hispanic or non-Latino ethnicity (1041/1137, 91.56%). In addition, 50.22% (571/1137) of respondents reported Democrat as their political affiliation, 91.47% (1040/1137) had health insurance, 67.37% (766/1137) went for regular annual checkups, and 42.22% (480/1137) had at least one chronic disease.

**Table 2:** Sociodemographic characteristics of the respondents by generation (N=1137).

Sociodemographic characteristic		Overall	Generation	
			Digital natives (n=571)	Digital immigrants (n=566)
Age (years), mean (SD)		44.2 (13.8)	32.5 (6.0)	56.0 (8.3)
**Gender, n (%)**				
	Male	574 (50.5)	288 (50.4)	286 (50.5)
	Female	563 (49.5)	283 (49.6)	280 (49.5)
**Marital status, n (%)**				
	Married	523 (46)	211 (37)	312 (55.1)
	Single	614 (54)	360 (63)	254 (44.9)
**Employment, n (%)**				
	Unemployed	299 (26.3)	142 (24.9)	157 (27.7)
	Employed	838 (73.7)	429 (75.1)	409 (72.3)
**Income (US $), n (%)**				
	<25,000	136 (12)	70 (12.3)	66 (11.7)
	25,000-34,999	106 (9.3)	55 (9.6)	51 (9)
	35,000-49,999	171 (15)	82 (14.4)	89 (15.7)
	50,000-64,999	140 (12.3)	86 (15.1)	54 (9.5)
	65,000-79,999	134 (11.8)	64 (11.2)	70 (12.4)
	80,000-99,999	124 (10.9)	71 (12.4)	53 (9.4)
	≥100,000	326 (28.7)	143 (25)	183 (32.3)
**Educational level, n (%)**				
	Lower than high school graduate	7 (0.6)	5 (0.9)	2 (0.4)
	High school graduate	149 (13.1)	81 (14.2)	68 (12)
	Some college or associate degree	351 (30.9)	167 (29.2)	184 (32.5)
	Bachelor’s degree	459 (40.4)	250 (43.8)	209 (36.9)
	Graduate degree or higher	171 (15)	68 (11.9)	103 (18.2)
**Race, n (%)**				
	American Indian or Alaska Native	8 (0.7)	7 (1.2)	1 (0.2)
	Asian	68 (6)	52 (9.1)	16 (2.8)
	Black or African American	97 (8.5)	41 (7.2)	56 (9.9)
	Native Hawaiian or other Pacific Islander	2 (0.2)	2 (0.4)	0 (0)
	White	931 (81.9)	445 (77.9)	486 (85.9)
	Some other race	31 (2.7)	24 (4.2)	7 (1.2)
**Ethnicity, n (%)**				
	Hispanic or Latino	96 (8.4)	67 (11.7)	29 (5.1)
	Not Hispanic or Latino	1041 (91.6)	504 (88.3)	537 (94.9)
**Political affiliation, n (%)**				
	Democrat	571 (50.2)	289 (50.6)	282 (49.8)
	Republican	566 (49.8)	282 (49.4)	284 (50.2)
**Do you go for an annual checkup regularly?, n (%)**				
	No	371 (32.6)	237 (41.5)	134 (23.7)
	Yes	766 (67.4)	334 (58.5)	432 (76.3)
**Do you have health insurance?, n (%)**				
	No	97 (8.5)	64 (11.2)	33 (5.8)
	Yes	1040 (91.5)	507 (88.8)	533 (94.2)
**Do you have at least one chronic disease?, n (%)**				
	No	657 (57.8)	395 (69.2)	262 (46.3)
	Yes	480 (42.2)	176 (30.8)	304 (53.7)
**Online health information search frequency, n (%)**				
	Frequent	615 (54.1)	279 (48.9)	336 (59.4)
	Infrequent	522 (45.9)	292 (51.1)	230 (40.6)

### Health Information Searches by Generation

Three 1-way ANCOVAs using 7 covariates (quality of OHI, trust in OHI, trust in health care institutions, OHI search skills, race, ethnicity, and political affiliation) were conducted to determine whether there were significant differences in the 3 types of OHIS between the 2 generations (digital natives and digital immigrants). Table 3 presents the ANCOVA results. The 2 generations differed significantly in the 3 types of OHIS behaviors. Specifically, digital natives engaged in significantly more (*P*<.001) searches for health wellness information compared with digital immigrants. In contrast, digital immigrants carried out considerably more searches for health guidance information (*P*<.001) and health management information (*P*=.001) compared with digital natives.

**Table 3:** Summary statistics of outcome variables as a function of 2 generations^a^.

Outcome variable	Generation			*F* test (*df*)		*P* value		η^2^
	Digital natives (n=571), mean (SD)	Digital immigrants (n=566), mean (SD)						
HWIS^b^	2.41 (0.75)	2.13 (0.82)	28.22 (1, 1128)		<.001		0.024	
HGIS^c^	0.76 (0.78)	1.02 (0.81)	29.11 (1, 1128)		<.001		0.025	
HMIS^d^	0.70 (0.66)	0.83 (0.61)	10.45 (1, 1128)		.001		0.009	
PHA^e^	1.94 (0.89)	1.94 (0.90)	0.04 (1, 1128)		.85		0.000	

^a^A total of 4 analyses of covariance were conducted using 7 covariates: information quality, trust in online health information, trust in health care institutions, search skills, race, ethnicity, and political affiliation.

^b^HWIS: health wellness information search.

^c^HGIS: health guidance information search.

^d^HMIS: health management information search.

^e^PHA: preventative health actions.

### Preventative Health Actions by Generation

A 1-way ANCOVA using generation as an independent variable and the same 7 covariates (quality of OHI, trust in OHI, trust in health care institutions, OHI search skills, race, ethnicity, and political affiliation) was conducted to determine whether there were significant differences in preventative health actions between the 2 generations. Table 3 presents the ANCOVA results. The 2 generations—digital natives and digital immigrants—did not differ significantly in their steps toward preventative health actions (*P*=.85).

We also tested whether OHI searches mediated the generational effects on preventative health actions and conducted 3 mediation analyses (one for each type of OHIS, namely, HWIS, HGIS, and HMIS). We selected the PROCESS Macro Model 4 for mediation analysis (simple mediation), with 20,000 bootstraps and 95% CIs with generation as an independent variable, preventative health actions as a dependent variable, OHI search types as the mediator, and 7 covariates (quality of OHI, trust in OHI, trust in health care institutions, OHI search skills, race, ethnicity, and political affiliation). The relationship between generation and preventative health actions was mediated (as the 95% CI did not include 0 for all 3 OHIS types) by each type of OHIS. Table 4 presents the mediation analysis results.

**Table 4:** Mediation analysis results^a^.

Indirect effect		Coefficient (SE)	Bootstrapping 95% CI
**HWIS^b^**			
	Total indirect effect	–0.08 (0.02)	–0.12 to –0.05
	Generation and HWIS	–0.25 (0.05)	–0.35 to –0.16
	HWIS and PHA^c^	0.31 (0.03)	0.25 to 0.38
**HGIS^d^**			
	Total indirect effect	0.08 (0.02)	0.05 to 0.11
	Generation and HGIS	0.26 (0.05)	0.17 to 0.36
	HGIS and PHA	0.29 (0.03)	0.23 to 0.36
**HMIS^e^**			
	Total indirect effect	0.06 (0.02)	0.02 to 0.11
	Generation and HMIS	0.12 (0.04)	0.05 to 0.20
	HMIS and PHA	0.52 (0.04)	0.44 to 0.59

^a^A total of 3 mediation analyses (PROCESS Macro Model 4) were conducted using 7 covariates: information quality, trust in online health information, trust in health care institutions, search skills, race, ethnicity, and political affiliation.

^b^HWIS: health wellness information search.

^c^PHA: preventative health actions.

^d^HGIS: health guidance information search.

^e^HMIS: health management information search.

### Age as a Predictor of Health Information Searches and Preventative Health Actions

To test the effect of age on OHIS types (HWIS, HGIS, and HMIS) and preventative health actions, we conducted 4 linear regression analyses using age as an independent variable, OHIS types (HWIS, HGIS, and HMIS) and preventative health actions as dependent variables, and 7 covariates (quality of OHI, trust in OHI, trust in health care institutions, OHI search skills, race, ethnicity, and political affiliation). Table 5 presents the linear regression results. Age was a significant negative predictor of HWIS behavior; as people aged, they searched for significantly less health wellness information (*P*<.001). In contrast, age was a significant positive predictor of HGIS and HMIS behaviors; as people aged, they searched for significantly more health guidance information (*P*<.001) and health management information (*P*=.003). However, age was not a significant predictor of preventative health actions (*P*=.48).

**Table 5:** Linear regression results.

		Unstandardized coefficient (SE)	*t* test	*P* value
**HWIS ^a,b^**				
	Age (years)	–0.01 (0.002)	–7.69	<.001
	Information quality	–0.04 (0.03)	–1.33	.18
	Trust in online health information	0.01 (0.03)	0.40	.69
	Trust in health care institutions	0.01 (0.02)	0.27	.78
	Search skills	0.01 (0.02)	0.62	.53
	Race	0.06 (0.02)	2.91	.004
	Ethnicity	–0.11 (0.09)	–1.29	.20
	Political affiliation	0.05 (0.05)	1.15	.25
	Constant	2.95 (0.21)	14.13	<.001
**HGIS^c,d^**				
	Age (years)	0.01 (0.002)	5.08	<.001
	Information quality	–0.04 (0.03)	–1.13	.26
	Trust in online health information	0.02 (0.03)	0.67	.51
	Trust in health care institutions	0.01 (0.02)	0.59	.56
	Search skills	–0.01 (0.02)	–0.64	.53
	Race	0.04 (0.02)	1.52	.13
	Ethnicity	0.03 (0.09)	0.32	.75
	Political affiliation	0.01 (0.05)	0.24	.81
	Constant	0.43 (0.22)	2.00	.045
**HMIS^e,f^**				
	Age (years)	0.004 (0.001)	2.96	.003
	Information quality	0.01 (0.03)	0.47	.64
	Trust in online health information	–0.06 (0.03)	–2.20	.03
	Trust in health care institutions	0.02 (0.02)	1.21	.23
	Search skills	–0.02 (0.02)	–0.99	.32
	Race	0.02 (0.02)	1.25	.21
	Ethnicity	0.08 (0.07)	1.11	.27
	Political affiliation	–0.01 (0.04)	–0.26	.79
	Constant	0.56 (0.17)	3.25	.001
**PHA^g,h^**				
	Age (years)	–0.001 (0.002)	–0.71	.48
	Information quality	–0.004 (0.04)	–0.10	.92
	Trust in online health information	–0.04 (0.04)	–1.24	.22
	Trust in health care institutions	–0.02 (0.02)	–0.78	.44
	Search skill	–0.04 (0.02)	–1.69	.09
	Race	0.02 (0.03)	0.77	.44
	Ethnicity	0.09 (0.10)	0.95	.35
	Political affiliation	–0.07 (0.05)	–1.27	.21
	Constant	2.24 (0.24)	9.37	<.001

^a^HWIS: health wellness information search.

^b^Model summary: *R*^2^=0.07; *F*_8,1128_=11.34; *P*<.001.

^c^HGIS: health guidance information search.

^d^Model summary: *R*^2^=0.03; *F*_8,1128_=4.00; *P*<.001.

^e^HMIS: health management information search.

^f^Model summary: *R*^2^=0.02; *F*_8,1128_=2.75; *P*=.005.

^g^PHA: preventative health actions.

^h^Model summary: *R*^2^=0.02; *F*_8,1128_=3.01; *P*=.002.

### Search Frequency as a Moderator

To examine whether the frequency of OHIS (frequent vs infrequent) moderated the effect of generation on OHIS type and preventative health actions, we conducted four 2-way ANCOVAs using generation, search frequency, and interaction term as independent variables and 7 covariates (quality of OHI, trust in OHI, trust in health care institutions, OHI search skills, race, ethnicity, and political affiliation). Table 6 presents the ANCOVA results. As expected, frequent OHI seekers, compared with infrequent OHI seekers, performed significantly more searches for all 3 types of health information (*P*<.001 for HWIS, HGIS, and HMIS) and engaged in significantly more preventative health behaviors (preventative health actions: *P*<.001). However, the interaction effects of generation and search frequency were not significant for all analyses. Thus, the frequency of OHIS did not moderate the relationship between generation and both type of OHIS and preventative health actions.

**Table 6:** Summary statistics of outcome variables as a function of 2 generations by online health information (OHI) search frequency^a^.

Outcome variables			Frequent, mean (SD)		Infrequent, mean (SD)		*F* test (*df*)		*P* value		η^2^
**HWIS^b^**											
	Generation	N/A^c^		N/A		45.24 (1, 1126)		<.001		0.04	
	OHI search frequency	N/A		N/A		100.99 (1, 1126)		<.001		0.08	
	Generation × OHI search frequency	N/A		N/A		2.18 (1, 1126)		.14		0.002	
	Digital natives	2.62 (0.62)		2.22 (0.82)		N/A		N/A		N/A	
	Digital immigrants	2.34 (0.72)		1.81 (0.86)		N/A		N/A		N/A	
**HGIS^d^**											
	Generation	N/A		N/A		18.37 (1, 1126)		<.001		0.02	
	OHI search frequency	N/A		N/A		200.91 (1, 1126)		<.001		0.15	
	Generation × OHI search frequency	N/A		N/A		0.59 (1, 1126)		.44		0.001	
	Digital natives	1.10 (0.78)		0.44 (0.64)		N/A		N/A		N/A	
	Digital immigrants	1.26 (0.78)		0.67 (0.73)		N/A		N/A		N/A	
**HMIS^e^**											
	Generation	N/A		N/A		3.68 (1, 1126)		.06		0.003	
	OHI search frequency	N/A		N/A		211.86 (1, 1126)		<.001		0.16	
	Generation × OHI search frequency	N/A		N/A		0.35 (1, 1126)		.56		0.000	
	Digital natives	0.97 (0.64)		0.45 (0.57)		N/A		N/A		N/A	
	Digital immigrants	1.03 (0.56)		0.53 (0.55)		N/A		N/A		N/A	
**PHA^f^**											
	Generation	N/A		N/A		2.25 (1, 1126)		.13		0.002	
	OHI search frequency	N/A		N/A		125.21 (1, 1126)		<.001		0.10	
	Generation × OHI search frequency	N/A		N/A		0.50 (1, 1126)		.48		0.000	
	Digital natives	2.22 (0.78)		1.68 (0.90)		N/A		N/A		N/A	
	Digital immigrants	2.19 (0.80)		1.57 (0.92)		N/A		N/A		N/A	

^a^A total of 4 analyses of covariance were conducted using 7 covariates: information quality, trust in OHI, trust in health care institutions, search skills, race, ethnicity, and political affiliation.

^b^HWIS: health wellness information search.

^c^N/A: not applicable.

^d^HGIS: health guidance information search.

^e^HMIS: health management information search.

^f^PHA: preventative health actions.

### Gender as a Moderator

To examine whether gender (male vs female) moderated the effect of generation on OHIS type (HWIS, HGIS, and HMIS) and preventative health actions, we conducted four 2-way ANCOVAs using generation, gender, and interaction term as independent variables and 7 covariates (quality of OHI, trust in OHI, trust in health care institutions, OHI search skills, race, ethnicity, and political affiliation). Table 7 presents the ANCOVA results. The interaction effect of generation and gender was significant only for HGIS (*P*=.02); among digital natives, female individuals, compared with male individuals, searched for significantly more health guidance information, whereas no significant differences among digital immigrants were observed between male and female individuals for HGIS. Moreover, the interaction effect of generation and gender was not significant for HWIS, HMIS, or preventative health actions. Thus, gender only moderated the relationship between generation and HGIS.

**Table 7:** Summary statistics of outcome variables as a function of 2 generations by gender^a^.

Outcome variables			Male, mean (SD)		Female, mean (SD)		*F* test (*df*)		*P* value		η^2^
**HWIS^b^**											
	Generation	N/A^c^		N/A		28.68 (1, 1126)		<.001		0.03	
	Gender	N/A		N/A		8.54 (1, 1126)		.004		0.01	
	Generation × gender	N/A		N/A		0.00 (1, 1126)		>.99		0.000	
	Digital natives	2.49 (0.73)		2.34 (0.77)		N/A		N/A		N/A	
	Digital immigrants	2.19 (0.85)		2.06 (0.80)		N/A		N/A		N/A	
**HGIS^d^**											
	Generation	N/A		N/A		29.71 (1, 1126)		<.001		0.03	
	Gender	N/A		N/A		12.18 (1, 1126)		<.001		0.01	
	Generation × gender	N/A		N/A		5.38 (1, 1126)		.02		0.01	
	Digital natives	0.63 (0.78)		0.90 (0.77)		N/A		N/A		N/A	
	Digital immigrants	0.99 (0.79)		1.05 (0.83)		N/A		N/A		N/A	
**HMIS^e^**											
	Generation	N/A		N/A		10.69 (1, 1126)		.001		0.01	
	Gender	N/A		N/A		10.51 (1, 1126)		.001		0.01	
	Generation × gender	N/A		N/A		1.65 (1, 1126)		.20		0.001	
	Digital natives	0.62 (0.66)		0.79 (0.66)		N/A		N/A		N/A	
	Digital immigrants	0.79 (0.60)		0.86 (0.61)		N/A		N/A		N/A	
**PHA^f^**											
	Generation	N/A		N/A		0.04 (1, 1126)		.85		0.000	
	Gender	N/A		N/A		1.13 (1, 1126)		.29		0.001	
	Generation × gender	N/A		N/A		0.69 (1, 1126)		.41		0.001	
	Digital natives	1.90 (0.89)		1.99 (0.88)		N/A		N/A		N/A	
	Digital immigrants	1.93 (0.91)		1.95 (0.89)		N/A		N/A		N/A	

^a^A total of 4 analyses of covariance were conducted using 7 covariates: information quality, trust in online health information, trust in health care institutions, search skills, race, ethnicity, and political affiliation.

^b^HWIS: health wellness information search.

^c^N/A: not applicable.

^d^HGIS: health guidance information search.

^e^HMIS: health management information search.

^f^PHA: preventative health actions.

### Presence of Chronic Diseases as a Moderator

To examine whether the presence of chronic diseases (yes vs no) moderated the effect of generation on OHIS types (HWIS, HGIS, and HMIS) and preventative health actions, we conducted four 2-way ANCOVAs using generation, presence of chronic diseases, and interaction term as independent variables and 7 covariates (quality of OHI, trust in OHI, trust in health care institutions, OHI search skills, race, ethnicity, and political affiliation). Table 8 presents the ANCOVA results. The interaction effects of generation and presence of chronic diseases were significant only for HWIS (*P*=.03). Specifically, digital natives with at least one chronic disease searched for more health wellness information than those without chronic diseases, whereas no significant differences among digital immigrants were observed between the presence or absence of a chronic disease for HWIS. Moreover, the interaction effects of generation and presence of chronic diseases were not significant for HGIS, HMIS, or preventative health actions. Thus, the presence of chronic diseases only moderated the relationship between generation and HWIS.

**Table 8:** Summary statistics of outcome variables as a function of 2 generations by presence of chronic diseases^a^.

Outcome variables		Chronic disease (no), mean (SD)	Chronic disease (yes), mean (SD)	*F* test (*df*)	*P* value	η^2^
**HWIS^b^**						
	Generation	N/A^c^	N/A	33.26 (1, 1126)	<.001	0.03
	Presence of chronic diseases	N/A	N/A	1.95 (1, 1126)	.16	0.002
	Generation × presence of chronic diseases	N/A	N/A	5.06 (1, 1126)	.03	0.004
	Digital natives	2.36 (0.77)	2.53 (0.70)	N/A	N/A	N/A
	Digital immigrants	2.15 (0.85)	2.11 (0.81)	N/A	N/A	N/A
**HGIS^d^**						
	Generation	N/A	N/A	7.83 (1, 1126)	.01	0.01
	Presence of chronic diseases	N/A	N/A	124.97 (1, 1126)	<.001	0.10
	Generation × presence of chronic diseases	N/A	N/A	0.89 (1, 1126)	.35	0.001
	Digital natives	0.58 (0.72)	1.16 (0.78)	N/A	N/A	N/A
	Digital immigrants	0.76 (0.79)	1.24 (0.77)	N/A	N/A	N/A
**HMIS^e^**						
	Generation	N/A	N/A	0.62 (1, 1126)	.43	0.001
	Presence of chronic diseases	N/A	N/A	112.81 (1, 1126)	<.001	0.09
	Generation × presence of chronic diseases	N/A	N/A	0.28 (1, 1126)	.60	0.000
	Digital natives	0.57 (0.65)	1.00 (0.60)	N/A	N/A	N/A
	Digital immigrants	0.62 (0.57)	1.00 (0.59)	N/A	N/A	N/A
**PHA^f^**						
	Generation	N/A	N/A	1.50 (1, 1126)	.22	0.001
	Presence of chronic diseases	N/A	N/A	14.88 (1, 1126)	<.001	0.01
	Generation × presence of chronic diseases	N/A	N/A	1.30 (1, 1126)	.26	0.001
	Digital natives	1.85 (0.88)	2.15 (0.86)	N/A	N/A	N/A
	Digital immigrants	1.86 (0.85)	2.01 (0.94)	N/A	N/A	N/A

^a^A total of 4 analyses of covariance were conducted using 7 covariates: information quality, trust in online health information, trust in health care institutions, search skills, race, ethnicity, and political affiliation.

^b^HWIS: health wellness information search.

^c^N/A: not applicable.

^d^HGIS: health guidance information search.

^e^HMIS: health management information search.

^f^PHA: preventative health actions.

## Discussion

### Principal Findings

In this study, we examined how the experiences with technology of 2 generations (digital natives and digital immigrants) affected their OHIS and preventative health behaviors using 7 control variables (information quality, trust in OHI, trust in health care institutions, search skills, race, ethnicity, and political affiliation). We found that digital natives searched more for health wellness information than digital immigrants, whereas digital immigrants searched more for health management and health guidance information than digital natives. However, after seeking OHI, the 2 generations did not differ significantly in their steps toward preventative health actions. We also found that OHI search types served as a mediator between generational differences and preventive health outcomes, whereas the search frequency of OHI did not moderate the relationship between generation and type of OHIS. In addition, gender only moderated the relationship between generation and HGIS; in contrast, the presence of chronic diseases only moderated the relationship between generation and HWIS. Further analysis (using age as a continuous measure) revealed that, as respondents’ ages increased, they sought significantly less health and wellness information but significantly more health management and health guidance information. However, age was not found to be a significant predictor of preventative health actions.

### Comparison With Prior Work

Previous research on OHIS has found that younger people are more prone to seeking OHI than older people [2-7]. The findings of this study are consistent with those of previous research indicating that younger generations (digital natives; aged between 18 and 42 years) are more likely to search for OHI than older generations (digital immigrants; aged ≥43 years). In addition, this study revealed generational differences in the types of OHI search behaviors.

In total, 3 types of OHIS (HWIS, HGIS, and HMIS) were used to analyze generational differences in search behavior. The findings revealed that digital natives (the younger generation) conducted more HWISs than digital immigrants (the older generation). The opposite was true of HGIS and HMIS—digital immigrants (the older generation) conducted more of these searches than digital natives (the younger generation). Some of these findings confirm previous research, whereas others contradict it. For example, research has shown that older adults tend to search for health information related to specific diseases, medications, and treatment guidance [9]. Similarly, this study found that digital immigrants conducted more HGI searches (namely, medication guidance and disease consulting) than digital natives. In addition, the results revealed that digital immigrants conducted more HMISs (such as managing health conditions and participating in online support groups) than digital natives. However, unlike previous research indicating that older adults tend to seek information related to nutrition and exercise [9], this study found that younger generations conducted more HWISs (namely, sports and fitness, nutrition and diet, and general health knowledge). These findings deviate from those of previous research, which shows that young adults seek health information online by joining social media groups and following pages relevant to their condition [44].

Previous findings have highlighted that OHIS is associated with positive health behaviors and health outcomes [85-91]. Although digital natives and digital immigrants did not differ in their preventative behaviors, this study found that the type of health information searches drove the effect of generational differences on preventive health outcomes by demonstrating the indirect link between OHIS and preventative health behaviors after retrieving all 3 types of OHI. This finding is significant as both generations reported engaging in preventive actions after seeking specific types of OHI. In addition, previous studies have established that the frequency of OHIS is higher among younger people [3,7,67]. However, the frequency of OHIS did not moderate the relationship between younger and older generations and types of OHI searches. The findings indicate that both frequent and infrequent OHI searchers from both generations in our sample conducted similar levels of 3 types of health information searches online, a departure from previous findings.

Furthermore, previous research has shown gender to be a significant predictor of OHIS behavior [92]. However, our analysis shows that gender only moderated the relationship between generation and searches for health guidance information (HGIS) but not searches related to health management information (HMIS) and health wellness information (HWIS). Similar to previous studies showing the association between people facing a health threat and health information–seeking behavior [93], we also found that the presence of at least one chronic disease moderated the relationship between generation and searches for health guidance information (HGIS) but not searches related to health management information (HMIS) and health wellness information (HWIS).

Although existing studies show that age, gender, ethnic background, political affiliation, and health conditions are significant predictors of OHIS behavior [3,7,92-96], there is a lack of research on whether generational differences affect the types of OHIS behavior or preventative behavior afterward [9]. Thus, our findings add to the OHIS behavior literature by illustrating that a single factor—generational cohorts that differ in their level of technology immersion—influences information-seeking and preventative health behaviors. This study furthers the understanding of OHI search behavior by differentiating between types of OHI search behavior across 2 generations.

### Limitations and Future Directions

This research is limited in several ways, indicating directions for future inquiry. First, we used stratified sampling to recruit 2 generations of American adults using an online data collection platform (Prolific). Specifically, we collected a stratified sample based on age, sex, and political affiliation using the United States as the location criterion. Although online data collection methods have many advantages, they are less likely to be used by those who do not have internet access. Thus, our findings are limited to digital natives and immigrants in this population sample. Future research could use in-person recruitment and focus groups to test the differences in OHIS and preventative health behaviors among different generations. Although we ensured an equal number of respondents in 2 digital generation groups (digital natives vs digital immigrants) with an even distribution of gender (male and female) and political affiliation (Republican and Democratic) and used 7 relevant control variables (information quality, trust in OHI, trust in health care institutions, search skills, race, ethnicity, and political affiliation) to show the effect of 2 generations on OHIS and preventative health behaviors, future research could use a more representative sample to test the validity of the relationships.

Second, this is the first study to our knowledge that has examined the differences in health information–seeking behaviors among older and younger Americans. This study focused on 2 generations that differ in their perception and use of technology: digital natives (born from 1980 onward) and digital immigrants (born before 1980). Although the use of 2 generational categories can simplify comparisons based on the ubiquity of technology in formative years, it has the potential to attenuate key variations within generational cohorts. Although our sample included nearly an equal number of digital natives and immigrants by age, sex, and political affiliation, most of our respondents were White, non-Hispanic or Latino Americans and skewed toward higher income brackets (nearly 40% made <US $50,000 a year; 413/1137, 36.32%) and educational levels (approximately 55% had a bachelor’s degree or higher; 630/1137, 55.41%). As such, our findings may overstate differences based on 2 generational cohorts, and future research could focus on recruiting more racial and ethnic minority groups and low-income individuals to assess the validity of our findings.

Third, we performed additional analyses using age as a continuous variable. Although using respondents’ age as a continuous variable shows results similar to those of categorizing them by generation, future research should test whether OHIS and preventative health behaviors vary among different generations, including microgenerations (such as Zillennials [14]). Thus, comparing how different generations in Western and non-Western countries differ in their OHIS and preventative health behaviors would be an exciting avenue to pursue. Moreover, technological exposure and norms [9] are likely to vary within 2 generations (digital immigrants and digital natives). Although we controlled for respondents’ self-reported online information search skills, other factors could be potential barriers that older adults face during OHIS, including self-efficacy and experience with information and communications technologies, perceived ease of use, and subjective norm perception [9,91,94].

Fourth, the statistical models used in the analyses did not account for some key variables. For example, previous research indicates that factors such as (but not limited to) health consumers’ perceived trust in health care professionals [97], ethnic background [3,94], and political ideology [95,96] are important predictors of OHIS and health behaviors. In addition, health information–seeking behavior has been shown to vary according to the perception of the credibility of information sources [34,35,64,65]. As our research did not account for these differences, the models used for the analyses may overstate differences based on generational cohorts.

Fifth, this study used self-reported OHIS and preventative health behavior measures, which might include biased responses, such as recall bias and social desirability bias [98,99]. Future research could benefit from using objective measures rather than relying on self-reports. For example, logged data of the OHI search history and daily diary study designs [100] would allow future researchers to measure types of health information searches and engagement in preventative health behaviors.

Finally, recent research suggests that some disadvantaged groups (older people and individuals of a lower socioeconomic status) lack the skills to use OHI and are often digitally marginalized [101]. Although this study did not explore digital marginalization, future research should address how lower technology skills might limit socially disadvantaged groups from participating in OHIS and managing their health.

### Conclusions

This study used a generational cohort lens to examine how older and younger generations differ in their OHI search and preventative health behaviors. This study provides insights into differences in OHI search behavior between the 2 digital generations, suggesting that, compared with digital immigrants, digital natives tend to search for more wellness information and are less likely to search for health management or guidance information. Furthermore, this research identified that the types of OHI searches mediated the generational effect on preventive health outcomes. Although the study of OHI search behavior is a well-established research domain, older adults’ OHI searches and preventative health behaviors are poorly understood [9]. As the older adult population and the demand for health care and online medical information increase, it is important to understand older adults’ OHIS and preventative health behaviors. This research could also serve as a foundation for future studies to understand how these results may apply across various population groups, including disadvantaged and digitally marginalized groups.

## Appendix

Multimedia Appendix 1 Survey questions.

## Abbreviations

ANCOVA: analysis of covariance
HGIS: health guidance information search
HMIS: health management information search
HWIS: health wellness information search
OHI: online health information
OHIS: online health information seeking

## Data Availability

The data sets generated during and analyzed during this study are available from the corresponding author on reasonable request.
