# Supplementary material for: Online Health Information Seeking and Preventative Health Actions: Cross-Generational Online Survey Study
Source: J Med Internet Res. 2024 Mar 11;26:e48977. doi: 10.2196/48977 (PMC10964147; doi:10.2196/48977)
Supplement: Multimedia Appendix 1 [file jmir_v26i1e48977_app1.docx]

**Multimedia Appendix 1: [Survey Questions]**

**General Instructions**

You are being invited to complete the following questionnaire to understand how people search for information online. Thank you for participating in this study. The data you provide will be used for marketing research.

There are no right or wrong responses, and your responses will be in no way associated with your identity. Please be honest with your answers. Please remember that your participation in this study is voluntary, you may stop at any time. Your responses will be kept strictly confidential. You need to be 18 years or older to complete this survey.

Please note:

(1) Please complete the study in one sitting.

(2) Please complete the study on the computer. Do NOT press the back/fresh button.

(3) Read the instructions closely on the current page before clicking CONTINUE to the next page.

The Institutional Review Board (IRB) at Bellarmine University has reviewed this study. If you have any questions about your rights as a research participant, you may call the IRB Office at 502-272-7963.

If you still wish to participate in this study, please click >> to CONTINUE.

-------- Page break ------------

**Frequency of OHIS**

How frequently did you search for online health information in the past 12 months?

*[only one option can be selected]*

- frequent
- infrequent
- never

-------- Page break ------------

**Types of Health-related Information**

Please indicate whether you have searched for the following health-related information during the past 12 months.

- Sports and fitness (Yes, No)
- Nutrition and diet (Yes, No)
- General health knowledge (Yes, No)
- Medication guidance (Yes, No)
- Disease consulting (Yes, No)
- Managing health conditions (Yes, No)
- Participating in an online support group (Yes, No)

-------- Page break ------------

**Preventative Health Actions**

- After seeking health information and finding the information, did your health behavior change for the better? (Yes, no)
- After seeking health information and finding the information, did you see a doctor? (Yes, no)
- After seeking health information and finding the information, did you monitor your health yourself for any changes? (Yes, no)

-------- Page break ------------

**Information quality of OHIS**

How would you rate the quality of health-related information on the Internet? (1 = very good quality; 7 = very poor quality)

**Trust in Internet health information**

How much would you trust health-related information on the Internet? (1 = a lot; 7 = not at all)

**Trust in United States healthcare institutions**

How much would you trust healthcare institutions in the US? (1 = a lot; 7 = not at all)

**Rating of searching skills**

Please rate your health-related information search skills on the Internet. (1 = very good, 7= very poor)

--- Page break ------------

**Please indicate your opinion on the following questions.**

- Do you go for annual checkup regularly? (Yes, no)
- Do you have health insurance? (yes/no)
- Do you have at least 1 chronic disease? (yes/no)

-------- Page break ------------

- What is your gender? (male, female)
- How old are you? (please enter your age in the below text box)
- Please indicate your annual household income in US dollars. (Less than $25,000, $25,000 - $34,999, $35,000 - $49,999, $50,000 - $64,999, $65,000 - $79,999, $80,000 - $99,999, $100,000 and more)
- What is your education level? (Less than high school graduate, High school graduate, Some college/Associate degree, Bachelor's degree, Graduate degree or higher)
- What is your race? (White, Black or African American, American Indian or Alaska Native, Asian, Native Hawaiian or Other Pacific Islander, Some Other Race)
- What is your ethnicity? (Hispanic or Latino, Not Hispanic or Latino)
- What is your political affiliation? (Democrat, Republican, Independent, Other)
- What is your employment status? (Unemployed, Students, Retired, Employed)
- What is your marital status? (Married, Unmarried, Divorced, Widowed)

-------- Page break ------------

**Attention Check**

If you are reading this, please do not answer this question and leave it blank. (1 = not at all true of me, 2 = slightly true of me, 3 = moderately true of me, 4 = very true of me, 5 = extremely true of me)

-------- Page break ------------

Thank you!

To receive payment, please type this access code into the box and click "Submit."

C1DKGP2S (The code for Digital Native (ages 18-42) and Democrat participants)

C14F3335 (The code for Digital Native (ages 18-42) and Republican participants)

C1E1124K (The code for Digital Immigrant (ages 43-99) and Democrat participants)

CUYQPYAT (The code for Digital Immigrant (ages 43-99) and Republican participants)
